# Quantification of cancer risk in glomerulonephritis

**DOI:** 10.1186/s12882-018-0828-2

**Published:** 2018-02-02

**Authors:** James Goya Heaf, Alastair Hansen, Gunnar Hellmund Laier

**Affiliations:** 1 Department of Medicine, Zealand University Hospital, Roskilde, Denmark; 2 Institute of Clinical Medicine, Herlev Hospital, University of Copenhagen, Copenhagen, Denmark; 3 Department of Production, Research and Innovation, Region Zealand, Sorø, Denmark

**Keywords:** Cancer, Glomerulonephritis, Epidemiology, Nephrotic syndrome, Uraemia, Proteinuria, Haematuria

## Abstract

**Background:**

The association of increased cancer risk with glomerulonephritis (GN) is well known, but controversy exists concerning which types of GN are involved, and the size of the association. A national registry survey was performed to assess the size of this association, and the temporal relationship of cancer diagnosis to GN diagnosis.

**Methods:**

All patients with biopsy-proven GN between 1985 and 2015 in Denmark were extracted from The Danish Renal Biopsy Registry and the National Pathology Data Bank. Incident cancer diagnoses between 10 years previous and 10 years subsequent to the GN diagnosis were extracted from the Danish Cancer Registry. Residence, birth and death data were obtained from the National Patient Register. Expected cancer incidence, classified according to cohort, age and sex were extracted from the Nordcan database.

**Results:**

Nine hundred eleven cancers were diagnosed in 5594 patients. Thirty five percent were prevalent at renal biopsy. Prevalence at biopsy was 5.5% (expected 3.1%), but incidence was not increased < 1 year before biopsy. Increased cancer rates were seen for GN forms: minimal change, endocapillary, focal segmental glomerulosclerosis, mesangioproliferative, membranous, focal segmental, membranoproliferative, proliferative, ANCA-associated vasculitis, lupus nephritis and unclassified. Increased cancer rates were seen for lung, prostate, renal, non-Hodgkin lymphoma, myeloma, leukaemia and skin. The increased incidence was mainly limited to − 1 to 1 year after biopsy, but skin cancer showed an increased risk over time. Some diagnoses showed an increase 5–10 years after biopsy. Incidence was raised for patients with uraemia and nephrosis, but less for proteinuria or haematuria. Cancers in patients < 45 years were rare. The risk of developing cancer 0–3 years after biopsy for patients 45–64 years varied from 7.3% (minimal change) to 15.8% (unclassified GN); > 64 years from 11.8 (endocapillary GN) to 20.3% (unclassified). The diagnosis with the highest risk was membranoproliferative GN (8.6 & 19.6%).

**Conclusions:**

Cancer rates are increased for many cancer and most GN diagnoses. Cancer screening for patients < 45 years and for patients without nephrosis or uraemia may not be necessary. The findings suggest that screening programs for specific GN diagnoses can be extended to other GN forms.

## Background

The association of cancer with glomerulonephritis (GN) is well known, being described as early as 1966 [1]. Detailed reviews of the subject have been published [2–6]. Most forms of glomerulonephritis have been implicated: minimal change disease (MCD), membranous nephropathy (MN), focal segmental glomerulosclerosis (FSGS), mesangioproliferative GN (MesPGN), membranoproliferative GN (also known as mesangiocapillary GN) (MPGN), anti-GBM disease (anti-GBMGN), and ANCA-related vasculitis (ANCAV). Similarly, a large number of cancer forms have been implicated, including lung, colorectal, stomach, renal, bladder, prostate, gynaecologic, breast, thymoma, Hodgkin’s lymphoma, non-Hodgkin’s lymphoma, and leukaemia. However, most of these associations are based on small series, with a limited number of cancer cases, which do not permit statistical analysis. The relationship of MN to cancer, primarily solid tumours is the best documented, with a number of large series. A metaanalysis [7] showed a prevalence of 10%, primarily lung, prostate, haematological and colorectal.

Few population-based studies are available. The association of proteinuria with increased later cancer risk is well documented [8–10]. In a Norwegian national study of 161 patients with MN between 1988 and 2003 [11], the cancer incidence after biopsy was 2.4%/year, 2.25 times the expected rate. Follow-up for 15 years did not seem to show a declining incidence. The largest population-based study was a Danish national study of all GN forms for the years 1985–96 [12]. This revealed 102 cancers in 1958 patients during a follow-up period of average 4.7 years, 1.93 times the expected value. Increased rates were seen for MCD, endocapillary GN (EndGN), MN, MesPGN, and unclassified GN. Cancers with increased rates were lung, skin, lymphatic and haematological, non-Hodgkin lymphoma, Hodgkin’s disease and leukaemia. Haematological malignancy in particular was 7–17 times more common than expected.

This database has now continued prospectively for 30 years, and permits a detailed study of cancer incidence, with accurate measurement of risk. We decided to repeat the analysis, with a follow-up period of 10 years. In addition, cancers diagnosed up to 10 years previous to biopsy were included, allowing prevalence rates to be assessed.

## Methods

All patients with biopsy-proven GN resident in Denmark between the years 1985–2014 were included. The great majority of patients were Caucasian.

The study was an observational study in epidemiology and followed the STROBE guidelines for reporting observational studies [13].

### Renal biopsies

The renal biopsy information was derived from two registries:

1. Danish Renal Biopsy Registry (DANYBIR). This registry recorded all biopsies performed in Denmark between 1985 and 1999 [14]. The reproducibility of the glomerular diagnosis has been investigated and found acceptable with a kappa value of 0.61 [15].
2. Since 2000 renal biopsy results have been registered by the National Pathology Data Bank (Patobank).

The following SNOMED diagnoses were included (SNOMED codes in parentheses): MCD, (M00100, combined with proteinuria or nephrosis: S65080, S67020, or S67550), endocapillary GN (EndGN, M46870), FSGS (M53341), MesPGN (M46862), MN (M68130), MPGN (M46842), proliferative GN (ProlGN, M46810), focal segmental proliferative GN (FSPGN, M46811), Focal GN (M46861), necrotizing and crescentic GN (also known as extracapillary GN) (NCGN, M46880), unclassified GN (M40000), anti-GBMGN (S67400), ANCAV (S76950), lupus nephritis (LN) (S38720). IgA GN was classified according to the light microscopy diagnosis, mainly MesPGN. Most cases of EndGN will have been infection-related GN. For each patient, only one biopsy was included, being the first biopsy with a GN diagnosis. Patients less than 15 years old were excluded. For biopsies with multiple GN diagnoses, the first mentioned diagnosis was chosen, with the following exceptions: anti-GBMGN and ANCAV were given first priority; MCD and unclassified GN were ignored in the presence of a more specific diagnosis. The presence in the biopsy of fibrosis (M49000-M49005) was noted.

Associated clinical diagnoses were registered (vide infra): Uraemia (SNOMED S65050, S65150, S65110 or ICD10 N03.x, N18.x), proteinuria (S65080 or N06.x), haematuria (65,070 or N02.x) and nephrotic syndrome (S67020, S67500, S67550 or N04.x).

### Cancer diagnoses

Using the patient national identity number, these patients were linked with all registered cancer diagnoses in the Danish Cancer Registry, which has been in existence since 1943. ICD-8 diagnoses were converted to the relevant ICD − 10 diagnoses. The following diagnosis groups were used: colon & rectum (C18.x -C21.x), lung (C26.x-C34.x), melanoma (C43.x), skin (not melanoma) (C44.x), breast (C50.0) gynaecologic (C54.x-C57.x), prostate (C61.x), renal (C64.x-c66.x), bladder (C67.x), Hodgkin lymphoma (C81.x), non-Hodgkin lymphoma (C82.x-c88.x), myeloma (C90.x), leukaemia (C91.x-C95.x), unclassified (all other Cxx.x diagnoses).

All diagnosis groups were incident at first diagnosis day, thereafter they were regarded as prevalent. “Any” cancer diagnosis was incident on the first day of the first cancer diagnosis, and was thereafter prevalent. Thus “Any cancer” prevalence will usually be less than the sum of individual cancer prevalences, since multiple cancers were not included.

### Cancer epidemiology

The Danish Cancer Registry exports all cancer data to the Nordic Cancer database (Nordcan) [16], which publishes yearly incident and prevalent data, stratified by diagnosis, 10-year age groups and sex. Data was extracted for three biopsy cohorts (1985–94, 1995–2004, 2005–2014), based on the results for 1990, 2000, and 2010 respectively.

### Patient data

Patient sex and birthday were calculated from the national identity number. Patients entered the study 10 years before the biopsy and were lost to follow-up (LTF) 10 years after, unless censored for death or emigration. Dates of immigration, emigration and death were extracted from the National Patient Registry (LPR). Clinical renal diagnoses (ICD-10 N03.x, N18.x, N02.x, N04.x, N06.x) were also extracted (vide supra).

### Calculations

Observation periods were: 10–5 years, 5–3 years, 3–1 years and 1–0 years before biopsy; 0–1, 1–3, 3–5, 5–10 years after biopsy. Analyses of prevalent rates at biopsy used the observation period 10–0 years before biopsy; overall cancer incidence − 10 to + 10 years. These observation periods were derived from the NORDCAN database.

The incidence of cancer increases with age. In order to measure this, for each year after biopsy, 10% of patients were assumed to move into the next 10-year age cohort.

For each observation period, the real observation period was often shorter than the defined, due to immigration, emigration or death. While this effect was minimal for periods before biopsy, it was substantial for periods after biopsy, particularly 3–10 years after biopsy, and was greater in the older age groups. The real average observation period for the relevant renal diagnosis and age group (15–44 years; 45–64 years; > 64 years) was used in calculations.

For incidence rates before biopsy, the rate of (by definition) survivable cancers was used. Thus, the incidence rate was measured by changes in prevalence rate.

Number of observed incident cancer episodes per time interval, and the expected incidence (%/year) were thus available. The observed incidence (%/yr) was defined as the number of observed cases divided by the average observation period. The expected number of cases in the time interval was defined as expected incidence multiplied by the average time period.

### Statistics

Statistical analysis was based on observed and expected cases. Confidence intervals and *p*-values for indirect standardized incidence ratios were calculated using the Poisson model for number of incident cancer cases given time at risk and population level incidence rates adjusted for age and calendar year.

## Results

The follow-up period was 6.4 ± 3.7 years; for patients followed for more than 5 years 7.5 ± 0.9 years. Nine hundred eleven cancers were diagnosed in 5594 patients.

### Glomerulonephritis and prevalent cancer

Three hundred thirty (36%) cancers diagnoses were prevalent at renal biopsy. Patient details and prevalence at biopsy are shown in Table 1. Prevalence at biopsy was 5.5% (expected 3.1%), but prevalence was only slightly increased < 1 year before biopsy, where it rose rapidly. Prevalence was raised for most GN diagnoses, with the exception of FSPGN, Focal GN, NCGN, anti-GBMGN and LN. Diagnoses with the highest prevalence were: unclassified, ANCAV, MPGN, FSGS and ProlGN.

**Table 1:** Patient age, sex, and cancer prevalence at diagnosis (observed vs. expected)

	No. patients	Age (yrs)	Female (%)	Patients with Cancer	Prevalence (%)	Expected prevalence (%)	Risk Ratio
Minimal change	428	43,3 ± 19	46	19	4,4	1.9	**2.4 (1.4–3.7)** ^**c**^
Endocapillary	137	43,4 ± 18	53	7	5,1	1.7	**3.0 (1.2–6.2)** ^**a**^
Focal segmental glomerulosclerosis	408	49,0 ± 17	34	27	6,6	2.7	**2.4 (1.6–3.5)** ^**c**^
Mesangioproliferative	1185	42,2 ± 17	36	36	3,0	1.7	**1,8 (1.2–2.5)** ^**b**^
Membranous	741	52,3 ± 17	41	37	5,0	3.2	**1.5 (1.1–2.1)** ^**a**^
Membranoproliferative	298	49,2 ± 17	50	22	7,4	2.4	**3.1 (1.9–4.7)** ^**c**^
Proliferative	113	43,4 ± 19	46	7	6,2	2.1	**2.9 (1.2–6.0)** ^**a**^
Focal segmental proliferative	507	47,3 ± 19	36	15	3,0	2.3	1,3 (0.7–2.1)
Focal	146	56,1 ± 17	41	6	4,1	3.1	1.3 (0.5–1.8)
Crescentic	610	58,4 ± 16	39	27	4,4	3.9	1.1 (0.7–1.6)
Unclassified	552	55,4 ± 18	41	82	14,9	3.0	**4.9 (3.9–6.1)** ^**c**^
Anti-GBMGN	92	55,5 ± 22	53	1	1,1	4.4	0.2 (0–1.4)
ANCA associated vasculitis	278	59,1 ± 16	38	21	7,6	4.2	**1,8 (1.1–2.5)** ^**a**^
Lupus nephritis unspecified	99	38,1 ± 17	77	1	1,0	1.5	0,7 (0–3.8)
Lupus nephritis all	422	36.4 ± 15	77	7	1.7	0.7	1.2 (0.5–2.5)
Any	5594	49.4 ± 18	41	330	5.5	3.1	**1,8 (1.4–2.1)** ^**c**^

Significant risk ratios in bold type

^a^:< 0.05; ^b^:< 0.01; ^c^:< 0.001

### Glomerulonephritis and cancer incidence

**Fig. 1:**
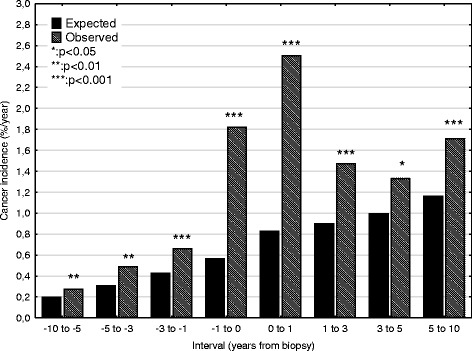
Observed and expected any cancer incidences for all biopsied

**Fig. 2:**
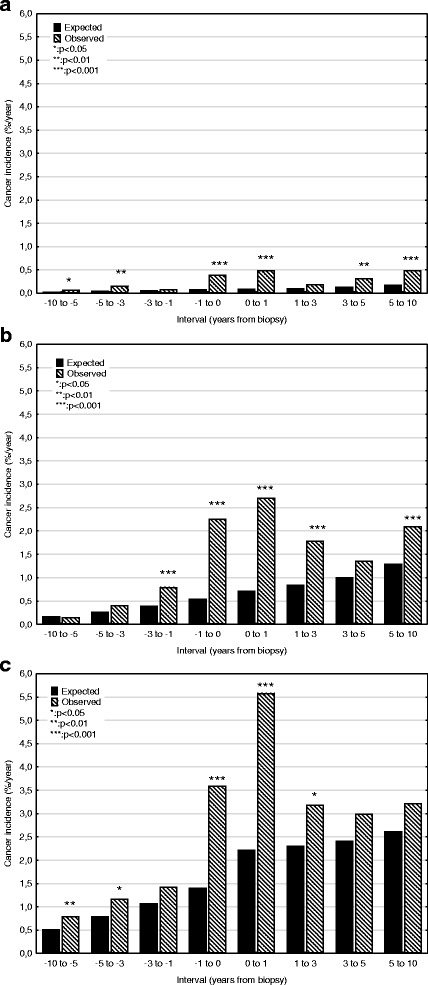
Observed and expected any cancer incidences according to age group. **a** 15–44 years; **b** 45–64 years; **c** > 64 years

**Fig. 3:**
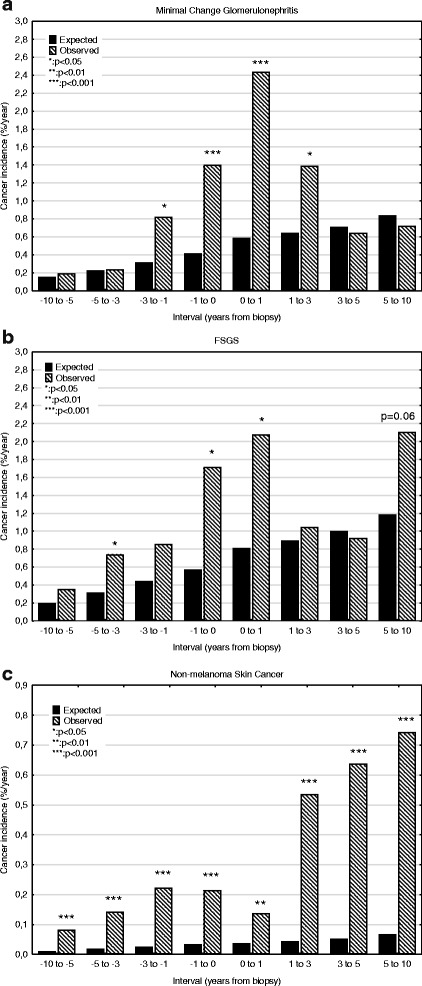
Patterns of observed cancer incidences according to time relationship to biopsy. **a** Usual pattern, with increase mainly limited to period −1 to 1 year after biopsy; **b** Increased incidence around biopsy date, with late increase (membranous nephropathy, FSGS, mesangioproliferative GN, proliferative, focal segmental proliferative GN; **c** increasing incidence during period of observation (focal segmental proliferative GN, non-melanoma skin cancer)

Incidence rates for GN are shown in Table 2 and Fig. 1. Incidence was slightly increased up to 1 year before biopsy, but then rose rapidly for the period of − 1 to 1 year, to a maximum of 2.5%/yr. The increase was related to age (Fig. 2): < 45 years 0.5%/yr., 45–64 years 2.7%/yr. and > 64 5.6%/yr. This pattern was present for MCD, EndGN, FSGS, MesPGN, MN, MPGN, ProlGN, LN, ANCAV and unclassified, but no increase was seen for focal GN, NCGN, or anti-GBMGN. About 40% of this increase was prevalent at biopsy, with the exception of MN, where only 20% was present. Incidence then fell, and was no longer significant at 3–5 years (Fig. 3, pattern a). This pattern was seen for the diagnoses MCD, EndGN, MPGN and unclassified. For some GN diagnoses (MesPGN, ProlGN, FSGS, MN), after an initial fall, an increased incidence was seen after 5 years (Fig. 3, pattern b). One GN diagnosis (FSPGN) showed a gradually increasing incidence (pattern C). The presence of fibrosis in the biopsy (14%) did not affect the cancer incidence.

**Table 2:** The association of renal diagnosis to observed and expected cancer incidence (%/year) grouped according to time interval relative to biopsy date

Time interval (years)		-10 to −5	−5 to −3	−3 to −1	−1 to 0	0–1	1–3	3–5	5–10	−10 to 10
Minimal change	Observed	0.19	0.24	0.82	1.40	2.44	1.39	0.64	0.72	0.73
	Expected	0.15	0.23	0.31	0.41	0.59	0.64	0.71	0.84	0.45
	Risk ratio	1.2 (0.3–3.2)	1.0 (0.1–3.8)	**2.6 (1.1–5.4)** ^**a**^	**3.4 (1.2–7.4)** ^**b**^	**4.1 (2.0–7.6)** ^**c**^	**2.2 (1.0–4.0)** ^**a**^	0.9 (0.2–2.3)	0.9 (0.4–1.6)	**1.6 (1.2–2.1)** ^**c**^
Endocapillary	Observed	0.15	0.36	1.09	1.46	3.32	0.50	1.51	0.29	0.72
	Expected	0.14	0.21	0.29	0.39	0.58	0.63	0.71	0.84	0.42
	Risk ratio	1.1 (0–6.1)	1.8 (0–9.9)	**3.8 (0.8–11.2)** ^**a**^	3.7 (0.4–13.4)	**5.8 (1.6–14.7)** ^**b**^	0.8 (0–4.4)	2.1 (0.3–7.7)	0.3 (0–1.9)	**1.7 (1.0–2.9)** ^**a**^
Focal segmental glomerulosclerosis	Observed	0.35	0.74	0.86	1.72	2.08	1.05	0.92	2.10	0.96
	Expected	0.20	0.32	0.44	0.57	0.81	0.89	1.00	1.19	0.57
	Risk ratio	1.8 (0.7–3.6)	**2.3 (0.9–5.1)** ^a^	1.9 (0.8–4.0)	**3.0 (1.2–6.2)** ^**a**^	**2.5 (1.1–5.0)** ^**a**^	1.2 (0.5–2.4)	0.9 (0.3–2.4)	**1.8 (0.9–3.0)** ^***p*** **= 0.06**^	**1.7 (1.3–2.2)** ^**c**^
Mesangio- proliferative	Observed	0.17	0.38	0.34	0.76	1.05	0.83	0.97	1.14	0.58
	Expected	0.12	0.19	0.26	0.36	0.50	0.56	0.63	0.77	0.38
	Risk ratio	1.4 (0.7–2.6)	**2.0 (0.9–3.9)** ^a^	1.3 (0.6–2.5)	**2.1 (1.0–4.1)** ^**a**^	**2.1 (1.0–3.8)** ^**a**^	1.5 (0.8–2.5)	1.5 (0.9–2.5)	**1.5 (1.0–2.1)** ^**a**^	**1.5 (1.2–1.8)** ^**c**^
Membranous	Observed	0.30	0.55	0.74	0.94	3.90	1.77	0.93	2.39	1.10
	Expected	0.22	0.33	0.47	0.62	0.91	0.99	1.10	1.30	0,65
	Risk ratio	1.4 (0.7–2.5)	1.6 (0.7–3.2)	1.6 (0.8–2.9)	1.5 (0.6–3.1)	**4.3 (2.8–6.3)** ^**c**^	**1.8 (1.1–2.8)** ^**a**^	0.8 (0.4–1.6)	**1.8 (1.3–2.6)** ^**c**^	**1.7 (1.4–2.0)** ^**c**^
Membrano- proliferative	Observed	0.48	0.34	0.68	3.04	3.81	2.24	1.45	1.05	1.18
	Expected	0.20	0.30	0.42	0.54	0.78	0.85	0.94	1.10	0.55
	Risk ratio	**2.4 (1.0–4.9)** ^**a**^	1.1 (0.1–4.0)	1.6 (0.4–4.1)	**5.6 (2.6–10.6)** ^**c**^	**4.9 (2.3–9.0)** ^**c**^	**2.6 (1.2–5.0)** ^**b**^	1.5 (0.4–4.0)	1.0 0.4–2.0)	**2.2 (1.6–2.8)** ^**c**^
Proliferative	Observed	0.53	0	0.88	1.78	1.92	0	0	2.95	0.87
	Expected	0.14	0.22	0.31	0.41	0.60	0.66	0.74	0.88	0.43
	Risk ratio	**3.7 (0.8–10.8)** ^**a**^	0 (0–7.6)	2.9 (0.3–10.4)	4.3 (0.5–15.5)	3.2 (0.4–11.5)	0 (0–3.2)	0 (0–3.5)	**3.4 (1.1–7.8)** ^**c**^	**2.0 (1.1–3.4)** ^**a**^
Focal segmental proliferative	Observed	0.16	0.20	0.49	0.79	1.14	1.29	1.50	1.76	0.74
	Expected	0.17	0.26	0.36	0.49	0.71	0.78	0.87	1.02	0.52
	Risk ratio	0.9 (0.3–2.4)	0.8 (0.1–2.8)	1.4 (0.5–3.2)	1.6 (0.4–4.2)	1.6 (0.5–3.7)	1.7 (0.8–3.0)	1.7 (0.8–3.2)	**1.7 (1.1–2.6)** ^**a**^	**1.4 (1.1–1.8)** ^**b**^
Focal	Observed	0	1.03	0.35	1.37	1.67	0.66	2.48	0.95	0.70
	Expected	0.29	0.44	0.61	0.78	1.14	1.23	1.34	1.55	0.75
	Risk ratio	0 (0–1.8)	2.3 (0.5–6.9)	0.6 (0–3.3)	1.8 (0.2–6.4)	1.5 (0.2–5.3)	0.5 (0–3.0)	1.9 (0.4–5.4)	0.6 (0.1–1.8)	0.9 (0.5–1.6)
Crescentic	Observed	0.23	0.49	0.49	1.31	1.00	1.88	2.49	2.28	0.91
	Expected	0.30	0.46	0.64	0.84	1.25	1.34	1.46	1.67	0.79
	Risk ratio	0.8 (0.3–1.6)	1.1 (0.4–2.3)	0.8 (0.3–1.9)	1.6 (0.7–3.1)	0.8 (0.3–1.9)	1.4 (0.8–2.4)	1.7 (0.9–2.9)	1.4 (0.8–2.1)	1.2 (0.9–1.4)
Unclassified	Observed	0.58	1.09	1.18	7.43	6.71	2.41	1.44	2.10	1.94
	Expected	0.28	0.43	0.59	0.77	1.14	1.22	1.33	1.52	0.74
	Risk ratio	**2.1 (1.2–3.3)** ^**a**^	**2.5 (1.3–4.4)** ^**b**^	**2.0 (1.1–3.4)** ^**a**^	**9.7 (6.9–13.1)** ^**c**^	**5,9 (4.0–8.3)** ^**c**^	**2,0 (1.2–3.1)** ^**b**^	1.1 (0.4–2.2)	1.4 (0.8–2.3)	**2.6 (2.2–3.1)** ^**c**^
Anti-GBM GN	Observed	0.00	0.54	0.00	0.00	1.22	3.11	0.00	2.93	0.6
	Expected	0.31	0.46	0.62	0.80	1.20	1.26	1.34	1.49	0.7
	Risk ratio	0 (0–2.6)	1.2 (0–6.6)	0 (0–3.3)	0 (0–5.1)	1.0 (0–5.7)	2.5 (0.5–7.2)	0 (0–2.8)	2.0 (0.4–5.8)	0.8 (0.4–1.6)
ANCA-associated vasculitis	Observed	0.50	0.54	1.08	1.80	2.96	1.90	2.43	2.48	1.37
	Expected	0.29	0.45	0.62	0.84	1.27	1.36	1.48	1.69	0.84
	Risk ratio	1.72 (0.7–3.5)	1.2 (0.2–3.5)	1.7 (0.6–3.8)	2.1 (0.7–5.0)	2.3 (0.9–4.8)	1.4 (0.6–2.9)	1.6 (0.7–3.1)	1.5 (0.8–2.5)	**1.6 (1.2–2.1)** ^**c**^
Lupus nephritis unspecified	Observed	0	0	0.51	0	1.14	1.71	2.40	0.75	0.60
	Expected	0.10	0.15	0.21	0.28	0.40	0.44	0.49	0.59	0.31
	Risk ratio	0 (0–7.9)	0 (0–12.7)	2.4 (0.1–13.6)	0 (0–13.3)	2.9 (0.1–15.9)	3.9 (0.5–14.0)	4.9 (0.6–17.6)	1.3 (0.3–3.7)	1.9 (0.9–3.7)
Lupus nephritis all	Observed	0.10	0.24	0.24	0.24	0.78	0.83	0.56	0.89	0.42
	Expected	0.09	0.14	0.19	0.25	0.34	0.38	0.43	0.52	0.26
	Risk ratio	1.1 (0.1–3.9)	1.7 (0.2–6.3)	1.2 (0.1–4.4)	0.9 (0–5.3)	2.3 (0.5–6.8)	2.2 (0.7–5.2)	1.3 (0.2–4.7)	1.7 (0.9–3.0)	**1.6 (1.1–2.3)** ^**a**^
Any	Observed	0.28	0.49	0.66	1.83	2.51	1.48	1.34	1.71	0.96
	Expected	0.20	0.31	0.43	0.57	0.83	0.90	1.00	1.17	0.58
	Risk ratio	**1.4 (1.1–1.7)** ^**b**^	**1.6 (1.2–2.1)** ^**b**^	**1.5 (1.2–1.9)** ^**c**^	**3.2 (2.6–3.9)** ^**c**^	**3.0 (2.5–3.6)** ^**c**^	**1.6 (1.4–2.0)** ^**c**^	**1.3 (1.1–1.7)** ^**a**^	**1.5 (1.3–1.7)** ^**c**^	**1.7 (1.5–1.8)** ^**c**^

Significant risk ratios in bold type

^a^:< 0.05; ^b^:< 0.01; ^c^:< 0.001

Ninety nine patients had unspecified lupus as primary diagnosis. A further 323 patients had lupus as secondary diagnosis. Their primary diagnoses were MesPGN (38%), MN (15%), MPGN (13%), ProlGN (9%) FS ProlGN (8%), End (7%), NCGN (5%) and unclassified (5%). Cancer risk was increased for all lupus patients combined, primarily after renal biopsy.

### Relationship of cancer type to incidence

Incidence rates for cancer are shown in Table 3. Pattern A was seen for the following cancer diagnoses (maximum incidence in %/yr. in brackets): lung (0.3), renal (0.3), non-Hodgkin lymphoma (0.2), myeloma (0.4), leukaemia (0.17), prostate (0.7), unclassified (0.5). Renal and unclassified cancers showed an increased incidence after 5 years. Unclassified cancers were mostly non-specific, but included 10 cases of gastric cancer and 10 cases of pancreatic cancer. Non-melanoma skin cancers followed a different pattern, with an increased incidence throughout the period of observation, rising from 0.1%/year 10 to 5 years prior to biopsy to 0.7 five to 10 years after (Fig. 3, pattern c). The late (5–10 years post-biopsy) overall increase in cancer incidence was almost entirely related to this increase in skin cancers. No increase in incidence was seen for melanoma, breast, gynaecologic, bladder, or Hodgkin lymphoma. The incidence of colorectal cancer was reduced. Other cancers associated with GN in the literature are shown in Table 4. These cancers were rare, not permitting statistical analysis.

**Table 3:** The association of cancer diagnosis to observed and expected cancer incidence (%/year) grouped according to time interval relative to biopsy date

Time interval (years)		−10 to −5	−5 to −3	−3 to −1	−1 to 0	0–1	1–3	3–5	5–10	−10 to 10
Colorectal	Observed	0.03	0.06	0.05	0.07	0.26	0.06	0.14	0.17	0.08
	Expected	0.05	0.09	0.12	0.17	0.22	0.24	0.27	0.33	0.16
	Risk ratio	0.5 (0,2–1.0)	0.7 (0.3–1.5)	**0.4 (0.2–0.9)** ^b^	0.4 (0.1–1.1)	1.2 (0.6–2.1)	**0.2 (0.1–0.5)** ^d^	0.5 (0.2–1.0)	**0.5 (0.3–0.9)** ^**c**^	**0.5 (0.4–0.6)** ^**d**^
Lung	Observed	0.00	0.03	0.04	0.20	0.32	0.26	0.17	0.23	0.10
	Expected	0.01	0.01	0.02	0.06	0.12	0.13	0.14	0.16	0.06
	Risk ratio	0.7 (0–3.9)	2.8 (0.6–8.2)	1.8 (0.5–4.6)	**3.1 (1.6–5.6)** ^**c**^	**2.7 (1.5–4.4)** ^**d**^	**2.0 (1.2–3.1)** ^**c**^	1.2 (0.6–2.3)	1.4 (0.9–2.1)	**1.6 (1.3–2.0)** ^**d**^
Melanoma	Observed	0.01	0.03	0.01	0.04	0.02	0.07	0.07	0.08	0.03
	Expected	0.02	0.02	0.03	0.03	0.03	0.03	0.03	0.04	0.03
	Risk ratio	0.7 (0.2–2.1)	1.3 (0.3–3.7)	0.3 (0–1.8)	1.1 (0.1–3.9)	0.6 (0–3.4)	2.3 (0.8–5.0)	2.1 (0.6–5.5)	2.1 (0.9–4.4)	1.2 (0.8–1.7)
Breast^a^	Observed	0.13	0.11	0.11	0.22	0.19	0.12	0.03	0.28	0.15
	Expected	0.09	0.12	0.17	0.18	0.18	0.19	0.20	0.22	0.15
	Risk ratio	1.4 (0.8–2.3)	0.9 (0.3–2.0)	0.6 (0.2–1.5)	1.2 (0.4–2.8)	1.0 (0.3–2.7)	0.6 (0.2–1.6)	**0.1 (0–0.8)** ^**b**^	1.2 (0.7–2.0)	1.0 (0.7–1.3)
Gynaecologic^a^	Observed	0.02	0.02	0.09	0.13	0.05	0.03	0.09	0.05	0.05
	Expected	0.04	0.05	0.06	0.07	0.09	0.09	0.10	0.11	0.07
	Risk ratio	0.4 (0.1–1.5)	0.4 (0–2.5)	1.4 (0.4–3.6)	1.7 (0.4–5.1)	0.5 (0–3.0)	0.4 (0–2.1)	1.0 (0.2–2.8)	0.5 (0.1–1.2)	0.7 (0.4–1.2)
Prostate^a^	Observed	0.04	0.08	0.12	0.49	0.74	0.22	0.20	0.27	0.19
	Expected	0.04	0.09	0.15	0.17	0.18	0.20	0.22	0.27	0.14
	Risk ratio	1.1 (0.5–2.3)	0.8 (0.3–1.9)	0.8 (0.4–1.6)	**2.9 (1.7–4.8)** ^**d**^	**4.2 (2.6–6.5)** ^**d**^	1.1 (0.5–2.0)	0.9 (0.4–1.9)	1.0 (0.6–1.6)	**1.3 (1.1–1.7)** ^**d**^
Renal	Observed	0.01	0.02	0.03	0.29	0.16	0.03	0	0.05	0.05
	Expected	0.004	0.01	0.01	0.01	0.02	0.02	0.02	0.02	0.01
	Risk ratio	2.0 (0.2–7.1)	2.9 (0.3–10.4)	3.0 (0.6–8.6)	**21.4 (12.2–34.8)** ^**d**^	**9.2 (4.0–18.2)** ^**d**^	1.8 (0.4–5.1)	0 (0–2.4)	**2.3 (0.9–4.7)** ^b^	**4.1 (2.9–3.5)** ^**d**^
Bladder	Observed	0.01	0.02	0.03	0	0.02	0.05	0.05	0.09	0.03
	Expected	0.02	0.03	0.04	0.05	0.06	0.07	0.08	0.09	0.05
	Risk ratio	**0** **.2** **(0–0.9** **)** ^**b**^	0.6 (0.1–2.0)	0.7 (0.1–1.9)	0 (0–1.3)	0.3 (0–1.8)	0.7 (0.1–2.1)	0.6 (0.1–1.8)	0.9 (0.4–1.9)	**0.5 (0.3–0.8)** ^**c**^
Hodgkin lymphoma	Observed	0	0.01	0.01	0	0.02	0	0.00	0.01	0.004
	Expected	0.002	0.002	0.002	0.003	0.003	0.003	0.003	0.003	0.003
	Risk ratio	0 (0–6.7)	3.9 (0.1–21.7)	3.7 (0.1–20.8)	0 (0–25.2)	6.3 (0.2–34.9)	0 (0–13.9)	0 (0–15.8)	3.2 (0.1–17.9)	1.6 (0.4–4.2)
Non-Hodgkin Lymphoma	Observed	0.01	0.02	0.03	0.16	0.17	0.03	0.06	0.06	0.04
	Expected	0.01	0.01	0.01	0.02	0.02	0.02	0.03	0.03	0.02
	Risk ratio	1.4 (0.3–4.2)	1.5 (0.2–5.5)	1.8 (0.4–5.3)	**8.1 (3.7–15.4)** ^**d**^	**7.3 (3.1–14.3)** ^**d**^	1.1 (0.1–4.0)	2.3 (0.6–5.9)	1.9 (0.7–4.1)	**2.5 (1.8–3.5)** ^**d**^
Myeloma	Observed	0.004	0.01	0.02	0.23	0.44	0.02	0	0.02	0.05
	Expected	0.001	0.003	0.01	0.01	0.01	0.01	0.01	0.01	0.01
	Risk ratio	3.1 (0.1–17.4)	3.0 (0.1–16.8)	3.6 (0.4–12.9)	**32.6 (17.3–55.7)** ^**d**^	**49.4 (31.0–74.8)** ^**d**^	2.2 (0.3–7.8)	0 (0–4.4)	1.6 (0.3–4.8)	**8.6 (6.3–11.6)** ^**d**^
Leukaemia	Observed	0.01	0.03	0.06	0.09	0.17	0.03	0.05	0.06	0.04
	Expected	0.01	0.02	0.02	0.03	0.04	0.05	0.05	0.06	0.03
	Risk ratio	0.8 (0.1–2.8)	1.7 (0.4–5.0)	**2.9 (1.2–6.0)** ^b^	**3.1 (1.0–7.3)** ^**b**^	**3.9 (1.7–7.7)** ^**c**^	0.7 (0.1–2.5)	1.0 (0.2–2.8)	0.9 (0.4–1.9)	**1.5 (1.0–2.0)** ^**b**^
Skin (non-melanoma)	Observed	0.08	0.14	0.22	0.21	0.14	0.54	0.64	0.74	0.27
	Expected	0.01	0.02	0.03	0.03	0.04	0.04	0.05	0.07	0.03
	Risk ratio	**7.8 (4.9–11.7)** ^**c**^	**7.4 (4.2–12.0)** ^**d**^	**8.6 (5.5–12.6)** ^**d**^	**6.4 (3.3–11.1)** ^**d**^	**3.6 (1.5–7.5)** ^**c**^	**12.1 (8.7–16.3)** ^**d**^	**12.1 (8.5–16.7)** ^**d**^	**10.9 (8.5–13.8)** ^**d**^	**8.7 (7.6–9.9)** ^**d**^
Unclassified	Observed	0.04	0.05	0.07	0.22	0.53	0.33	0.25	0.43	0.17
	Expected	0.04	0.05	0.07	0.11	0.19	0.20	0.22	0.25	0.12
	Risk ratio	1.1 (0.6–2.0)	1.1 (0.4–2.4)	1.0 (0.4–2.0)	**1.9 (1.0–3.3)** ^**b**^	**2.8 (1.9–4.1)** ^**d**^	**1.6 (1.1–2.4)** ^**b**^	1.1 (0.7–1.8)	**1.7 (1.2–2.3)** ^**c**^	**1.5 (1.2–1.7)** ^**d**^
Any not skin	Observed	0.20	0.35	0.46	1.63	2.37	0.99	0.77	1.06	0.71
	Expected	0.19	0.30	0.41	0.55	0.80	0.86	0.95	1.10	0.55
	Risk ratio	1.0 (0.8–1.3)	1.2 (0.8–1.6)	1.1 (0.8–1.5)	**2.9 (2.6–3.4)** ^**d**^	**3.0 (2.5–3.6)** ^**d**^	1.1 (0.9–1.4)	0.8 (0.6–1.1)	1.0 (0.8–1.1)	**1.3 (1.2–1.4)** ^**d**^

Significant risk ratios in bold type

^a^: relevant sex only. ^b^:< 0.05; ^c^:< 0.01; ^d^:< 0.001

**Table 4:** Individual cases of other cancers described in the literature as related to GN [3, 4]

Renal Diagnosis	Stomach	Pancreas	Liver	Sarcoma	Brain	Thymus
Minimal Change	0^a^	0^a^	1	0^a^		0^a^
FSGS	2^a^	2^a^		1^a^	0^a^	0^a^
Mesangioproliferative	4	2	2			0^a^
Membranous	1^a^	2^a^	1^a^	1^a^	1^a^	
Membranoproliferative	0^a^	1^a^	1			
Focal segmental proliferative		3			1	
Crescentic	1^a^		1^a^			0^a^
Unclassified	4		2			
ANCA vasculitis	2					

^a^ associations described in the literature

### Relationship of cancer incidence to Nephrological diagnosis

Clinical data was available was available for 4339 (78%) of biopsies: uraemia (62%), nephrotic syndrome (30%), proteinuria (15%) and haematuria (6%). Most of the increased risk was related to the diagnoses uraemia and nephrotic syndrome. Patients with haematuria had an overall increase risk (RR 1.5 (1.0–2.2), *p* < 0.05), but this was restricted to a late increase (5–10 years RR 3.2 (1.7–5.5), *p* < 0.001). Similarly, the overall relationship between proteinuria and cancer (RR 1.4 (1.1–1.8) was only apparent after 1 year post-biopsy.

### Relationship of specific Glomerulonephritis diagnoses with cancer diagnosis

Cancer incidence was only slightly increased up to 1 year prior to biopsy. In order to evaluate the specific relationship between GN and cancer, the number of expected cases between − 1 to + 10 years after biopsy was subtracted from the observed number. The total number of excess cases (observed minus expected) was 378. Distributions of excess cancers by GN and cancer diagnoses are shown in Tables 5 and 6, along with common associations in the literature. Considerable differences between observed and previously described associations were seen. With the exception of skin cancer which showed a generally increased risk for most cancer types, the number of cases per diagnosis was too small for statistical analysis, and clear patterns were not observed.

**Table 5:** Distribution of observed minus expected cases between −1 and 10 years after biopsy according to GN diagnosis (%)

Renal Diagnosis	Number	Lung	Melanoma	Breast	Gynaecologic	Prostate	Renal	Bladder	Non-Hodgkin	Myeloma	Leukaemia	Unclassified	Skin	Typical cancers in the literature
Minimal change	27	11			6				13	10		7	41	Renal, lung, colorectal, thymoma, Hodgkin’s
Endocapillary	6	37			11	24			14	15				
FSGS	28	21	10			5				6		21	31	Renal, thymoma
Mesangio-proliferative	53	12					9			12		21	45	Renal, lung, unclassified
Membranous	51	17	6			10					5	21	36	Renal, gastric, prostate, lung, unclassified
Membrano-proliferative	33	12							20	9	10	8	37	Renal, lung, leukaemia, unclassified
Proliferative	9	6		7				8	10			24	45	
Focal segmental proliferative	29				9	23						11	51	
ANCA vasculitis	26	6				10			5		11	7	61	Skin, leukaemia, bladder
Lupus (all)	17			9						11		21	58	Hodgkin, non-Hodgkin, myeloma, lung, leukaemia
Unclassified	92					5	25		9	23		16	17	

GN diagnoses with significantly raised incidence only. Negative figures excluded for this analysis. Only figures > 5% shown

**Table 6:** Distribution of 378 observed minus expected cases between −1 and 10 years after biopsy according to cancer diagnosis (%)

Cancer Diagnosis	Number	MCD	End	FSGS	MesPGN	MN	MPGN	FSPGN	Focal	NCGN	Unclassified	ANCAV	Typical GN in the literature [4]
Lung	37	8	6	16	17	23	11			6			MN, MCD, MPGN, MesPGN, FSGS, NCGN
Prostate	33					16		20		28	15	8	MCD, NCGN
Renal	30				16						78		NCGN, MesPGN, MCD, FSGS, MPGN
Non-Hodgkin	22	15					30				36	6	MN, MCD, MPGN, FSGS, NCGN
Myeloma	37	7		5	17		8				55		
Leukemia	15					16	23	10	10		22	19	MN, MCD, MPGN, FSGS, NCGN
Skin (non-melanoma)	152	7		6	16	12	8	10		12	11	10	MN
Unclassified	56			11	20	19	5		6		26		

Cancer diagnoses with significantly raised incidence only. Negative figures excluded for this analysis. Only figures > 5% shown. MCD: minimal change disease

*MN* membranous nephropathy, *FSGS* focal segmental glomerulosclerosis, *MesPGN* mesangioproliferative GN, *MPGN* Membranoproliferative GN, *FSPGN* focal segmental proliferative GN, *NCGN* necrotizing and crescentic GN

**Fig. 4:**
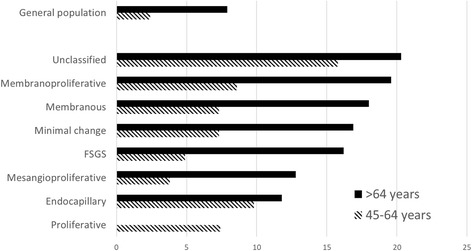
Risk of de novo cancer 0–3 years after biopsy by age

Assuming that all cancers occurring from 0 to 3 years after biopsy are diagnosable by invasive means at biopsy, the expected risk of different cancer forms during this period, grouped according to GN diagnosis, are shown in Table 7 and the overall incidence in Fig. 4.

**Table 7:** Risk of new cancer diagnosis 0–3 years after renal biopsy (%). A: 15–44 years; B: 45–64 years; C: > 64 years

Renal Diagnosis	Number	Colon	Lung	Melanoma	Breast^a^	Gynaecologic^a^	Prostate^a^	Renal	Bladder	Hodgkin	Non-Hodgkin	Myeloma	Leukemia	Unclassified	Skin	Any	Any no skin
A. 15–44 years																	
Total cancer cases		0	0	1	2	0	0	1	0	1	0	4	0	8	3	20	17
Minimal change	238			0.4						0.4				0.4		1.3	1.3
Endocapillary	85											1.3				1.3	1.3
FSGS	169													0.7		0.7	0.7
Mesangioproliferative	676											0.2		0.3		0.5	0.5
Membranous	253				0.7										0.4	0.9	0.4
Membranoproliferative	123											1.8			0.9	2.7	1.8
Proliferative	62																
Focal segmental proliferative	232													0.5		0.5	0.5
Focal	38																
Crescentic	110														1.0	1.0	
Unclassified	147							0.7						1.5		2.2	2.2
Anti-GBM GN	28													3.8		3.8	3.8
ANCA-associated	51																
Lupus unspecified	72				1.6											1.5	1.5
B. 45–64 years																	
Total cancer cases		7	16	2	2	0	11	7	1	0	3	11	4	24	21	108	87
Minimal change	119						1.5					1.8		2.7	1.8	7.3	5.5
Endocapillary	26		4.9								4.3					9.8	9.8
FSGS	162		1.4	0.7			0.9							0.7	1.4	4.9	3.5
Mesangioproliferative	367	0.3	0.6					0.3						0.9	1.6	3.8	2.2
Membranous	293	0.8	1.9	0.4	1.0		1.6	0.4					1.1	0.8	0.4	7.3	6.9
Membranoproliferative	112		2.1								1.0	1.1		1.1	3.2	8.6	5.4
Proliferative	32										3.4			3.7		7.4	7.4
Focal segmental proliferative	166	0.7					1.9							0.7	2.0	4.7	2.7
Focal	56	2.2	2.2													2.2	2.2
Crescentic	252	0.5			1.0		0.6							2.0	0.5	3.9	3.4
Unclassified	203	0.6	1.2				0.8	3.0	0.6			4.3		4.3	1.2	15.8	14.6
Anti-GBMGN	24						8.3									5.2	5.2
ANCA-associated	115		1.0				1.4						0.9	1.0	2.0	5.9	3.9
Lupus unspecified	18											7.0				7.0	7.0
C: > 64 years																	
Total cancer cases		10	19	4	4	2	20	3	3	0	7	9	6	22	25	129	105
Minimal change	71	3.4	1.7		2.9	2.9					1.7			1.7	5.1	16.9	11.8
Endocapillary	26						6.7							5.9	0.0	11.8	11.8
FSGS	77		4.9	1.6			1.7							4.9	3.2	16.2	13.0
Mesangioproliferative	142		2.0				2.2					3.0		3.9	3.0	12.8	9.9
Membranous	195	1.4	4.1	0.7	1.5		3.9				0.7		0.7	4.8	2.8	18.0	15.2
Membranoproliferative	63		2.5								2.5		7.4	0.0	7.4	19.6	12.3
Proliferative	19													0.0	0.0	0.0	0.0
Focal segmental proliferative	109		1.3	1.3		2.3	4.6							1.3	0.0	9.2	9.2
Focal	52	3.3													3.3	6.5	3.3
Crescentic	248	0.7	1.4	0.7	1.0		0.7		0.7				0.7	0.7	2.7	8.2	6.1
Unclassified	202	1.6	0.8		1.1		2.8	2.3	0.8		2.3	4.7	0.8	2.3	2.3	20.3	17.9
Anti-GBMGN	40		9.8													9.8	9.8
ANCA-associated	112	2.7					5.3		1.3		1.3			0.0	2.7	13.4	10.7
Lupus unspecified	9													24.3	0.0	24.3	24.3

^a^:Relevant sex only

## Discussion

The present study is the largest study of the relationship between GN and cancer, and permits a quantitative assessment of cancer risk for common forms of GN and cancer, and the temporal relationship between the two diseases. A further strength is that incidence was compared with the expected age- and sex-adjusted incidence in the general population. It confirms the increased cancer incidence seen in the previous study using this database [12] for MCD, EndGN, MN, MesPGN and unclassified GN. FSGS, ProlGN FSPGN, ANCAV and LN ratio were added to the list but no increase was seen for focal GN, NCGN, or anti-GBMGN. These findings are in general accord with the literature [2–6]. IgA GN and Henoch-Schönlein purpura were not specifically studied in this paper, but most of these patients will have had MesPGN.

As in the previous study, cancers with increased rates were lung, skin, non-Hodgkin lymphoma, and leukaemia. However increased rates for colorectal cancer and Hodgkin’s lymphoma could not be confirmed. Myeloma, renal, prostate and unclassified tumours were added to the list. These differences may simply due to chance given the large number of comparisons. Most of these findings have previously been described. However, the study does not support previously described relationships to e.g. Hodgkin’s lymphoma [7, 17], colorectal [7] and gastric cancer [3]. Some of these findings may be due to a type 2 statistical error due to relative paucity of the tumours. For instance, the lack of any cases of thymoma preclude any contribution to its well-documented role in GN [18]. Perhaps the most surprising finding is that non-melanoma skin tumours were the most common cancer form for several GN diagnoses. Indeed, the finding is probably an underestimate, in that cancers were considered prevalent at first diagnosis; most skin cancers can be expected to be definitively cured shortly after diagnosis. This observation has received little previous attention.

About 40% of excess cancers were present at biopsy, except for MN, where the figure was 20%. This is in accordance with the metaanalysis of MN by Leeaphorn et al. [7].

An epidemiological study can contribute little to a discussion of aetiology. Possible explanations include cancer causing GN, GN causing cancer due to the immunosuppressive effects of nephrosis and uraemia, immunosuppressive effects of GN therapy, or both being caused by a third factor. It is probable that the relationship is partly coincidental: an investigation for cancer may by chance reveal an undiscovered GN and vice versa [19]. This possibility is particularly relevant for renal cancer and myeloma, where renal biopsy may be included in the routine diagnosis of these diseases. Implementation of cancer screening for GN, which is common for MN will have hastened the diagnosis of subclinical cancers. However, if a renal biopsy resulted in a major overall shortening of the time to cancer diagnosis, one would expect that incidence for that cancer form would fall to below expected for the period 1–3 years after biopsy. Finally, the increased rate may be due to the carcinogenic effects of GN treatment. However, the effect of therapy is unlikely to have played a role in the 36% of cancers that were present at biopsy. Only five diseases, MesPGN, proliferative, FSGS, FSPGN and MN showed a late rise in incidence. While FSGS treatment is rarely carcinogenic, carcinogenic therapy, e.g. azathioprine and cyclophosphamide is common in MesPGN, MPGN, anti-GBMGN, lupus GN and ANCAV. These observations do not exclude the possibility that initiation of immunosuppressive therapy might cause the rapid progression of an already present sub-clinical cancer to clinical disease during the first year of treatment. The lack of a major association between cancer and non-nephrotic proteinuria or haematuria suggests that cancer is mainly associated with severe renal disease. Similarly, if one assumes that disease diagnosis time point is associated with disease severity and/or accelerating activity, the close temporal conjunction of the cancer and GN diagnoses suggest that any aetiological common agent is proportional to disease severity. One can speculate that the late rise in cancer incidence for patients with proteinuria and haematuria is thus caused by progression of the renal disease to more severe forms.

The association of specific cancers to specific GN diagnoses are shown in Tables 5 and 6. These are at some variance with the literature. There are well-known associations between both Hodgkin lymphoma [20] and thymoma [18] and MCD; this paper cannot contribute to this discussion due to too few patients. Other well recognized associations are solid tumours and MN [3], chronic lymphocytic leukemia and MPGN [21], and renal cell carcinomas and IgA nephropathy [22]. Possible aetiological mechanisms for these relationships include tumour-antibody production against shed tumour antigens (solid tumours and MN) [19]; putative circulating factor secreted by T lymphocytes, inducing glomerular cell cytoskeleton degeneration (Hodgkin lymphoma and MCD) [23]; B-cell production of either a cryoglobulin or M-component (chronic lymphocytic leukemia and MPGN) [24]. We found more non-specific relations between tumour type and GN diagnosis. This is not entirely unexpected: Lien et al. also found substantial variation in the linkage between GN form and cancer type [5]. There are several possible explanations for the discrepancies. The most common form of cancer was non-melanoma skin cancer. Since most of these cancers are cured at diagnosis, previous studies may have missed these cases. The actual numbers of cancers in each published series will be small, so considerable variation in relative incidence must be expected. Even in the present large study, the number of non-skin cancers per GN diagnosis only varied between 6 and 33. In contrast to other studies, where all cases of cancer were described, Tables 5 and 6 only study the relationship to excess tumours over and above the expected; this will tend to reduce the contribution of common cancers. We suggest therefore that our figures are more accurate than other studies; however, this question can only be resolved by a metaanalysis. It may even be possible that GN has a non-specific carcinogenic effect (or vice versa), and that previous associations with specific diagnoses rest upon an overinterpretation of small series. The practical consequence of these findings is that the presence of most GN forms should result in a generally increased non-specific suspicion of cancer.

The distribution of cancers being diagnosed 0–3 years after renal diagnosis are shown in Table 7 and Fig. 4. Assuming that most of these cancers can be diagnosed at biopsy, this table has practical significance. Cancer screening for MN has received considerable attention. Some authors [6, 25] recommend extensive screening including gastroscopy, colonoscopy, gynaecological investigation, ultrasonic renal scanning and CT thorax, while others do not advise other than standard age-specific screening in the absence of specific symptoms or objective findings. The KDIGO guidelines recommend specific malignancy screening for MN, FSGS and IgA GN, but not other forms of GN. The present study suggests that a more general suspicion of cancer should exist for other forms of GN. Patients under 45 years, and patients without uraemia or nephrotic syndrome do not appear to need further investigations. Otherwise measurement of myeloma protein, prostate specific antigen and a peripheral blood examination should be standard. An ultrasonic renal scan will usually be routine in connection with a renal biopsy. A CT thorax should be performed for smokers. Patients should be informed of an increased skin cancer risk and advised to reduce exposure to sun. Regular dermatological assessments may be beneficial. The present study does not suggest that there are indications for gastroscopy, coloscopy, gynaecological investigation or mammography, other than normal age-specific indications. Whole-body PET-CT or MR scans can be used for non-Hodgkin lymphoma and other rare malignancies, primarily for the GN diagnoses MPGN, MCD and unclassified GN. Figure 4 shows the risk of de novo cancer diagnoses after renal diagnosis, and can be used as a guideline for investigational programs.

A number of criticisms to this study can be raised. The study is limited to a small country, and the results may not be applicable to other populations. As this was a registry-based study, important clinical information was unavailable, such as the presence of HIV, hepatitis C and concurrent immunosuppressive therapy, which all lower immune tolerance. Biopsies were evaluated at different centres by different pathologists; some intra- and inter-individual differences in diagnoses will have been unavoidable. IgA nephropathy was not studied specifically; most of these patients will have been classified as MesPGN. MPGN is a mixed morphological category which could contain C3GN, cryoglobulinaemic GN, and IgA nephropathy. Similarly, LN has a number of light microscopy patterns. We have attempted to avoid this issue for LN by providing analyses both for all LN and for morphological subgroups. No data concerning potentially carcinogenic therapy were available. These problems document some of the limitations of registry-based research.

All cancers were assumed to be prevalent at first diagnosis, due to the difficulty of differentiating repeat registrations of the same cancer, cancer recurrence and de novo disease. Thus, the incidence rates presented here will be an underestimate of the true incidence, particularly for skin tumours (vide supra). While the study presents probably the largest number of patients and cancers addressing this question, sub-group analyses still often contain small numbers of cases, with large confidence intervals, and possibility of type 2 statistical errors. Similarly, the low incidence of rare tumours prevents meaningful conclusions for these. However, for more common forms of cancer, and cancer incidence generally, the study presents useful information for guiding physicians in screening practice.

## Conclusions

Cancer rates are increased for many cancer and most GN diagnoses. This study presents the first systematic quantification of this risk. The increased incidence is mainly confined to 1 year prior to 1 year after the renal diagnosis. Some GN diagnoses demonstrate a rise in cancer 5 years after diagnosis, which may be related to possible carcinogenic therapy. Most GN forms should result in a generally increased non-specific suspicion of cancer. Cancer screening for patients < 45 years and for patients without nephrosis or uraemia may not be necessary. This study presents useful information for guiding physicians in screening practice for common forms of cancer.

## Abbreviations

ANCAV: ANCA-associated vasculitis
Anti-GBMGN: Anti-glomerular basal membrane antibody glomerulonephritis
EndGN: Endocapillary glomerulonephritis
FSGS: Focal segmental glomerulosclerosis
FSPGN: Focal segmental proliferative glomerulonephritis
GN: Glomerulonephritis
LN: Lupus nephritis
MCD: Minimal change disease
MesPGN: Mesangioproliferative glomerulonephritis
MN: Membranous nephropathy
MPGN: Membranoproliferative glomerulonephritis
NCGN: Necrotizing and crescentic glomerulonephritis
ProlGN: Proliferative glomerulonephritis
